# Antimicrobial Effectiveness of Innovative Photocatalysts: A Review

**DOI:** 10.3390/nano12162831

**Published:** 2022-08-17

**Authors:** Giusy Lofrano, Francesca Ubaldi, Luisa Albarano, Maurizio Carotenuto, Vincenzo Vaiano, Federica Valeriani, Giovanni Libralato, Gianluca Gianfranceschi, Ilaria Fratoddi, Sureyya Meric, Marco Guida, Vincenzo Romano Spica

**Affiliations:** 1 Department of Movement, Health and Human Sciences, University of Rome Foro Italico, Piazza Lauro De Bosis, 15, 00135 Rome, Italy; giusy.lofrano@uniroma4.it (G.L.); f.ubaldi@studenti.uniroma4.it (F.U.); gianluca.gianfranceschi@uniroma4.it (G.G.); vincenzo.romanospica@uniroma4.it (V.R.S.); 2 Department of Biology, University of Naples Federico II, Via Vicinale Cupa Cintia 26, 80126 Naples, Italy; luisa.albarano@unina.it (L.A.); giovanni.libralato@unina.it (G.L.); marco.guida@unina.it (M.G.); 3 Department of Industrial Engineering, University of Salerno, Via Giovanni Paolo II, 132, 84084 Fisciano, Italy; mcarotenuto@unisa.it (M.C.); vvaiano@unisa.it (V.V.); 4 Department of Chemistry, Sapienza University of Rome, Piazzale Aldo Moro 5, 00185 Rome, Italy; ilaria.fratoddi@uniroma1.it; 5 Department of Environmental Engineering, Tekirdag Namik Kemal University, Corlu 59860, Turkey; smeric@nku.edu.tr

**Keywords:** pathogens, photocatalysts, disinfection

## Abstract

Waterborne pathogens represent one of the most widespread environmental concerns. Conventional disinfection methods, including chlorination and UV, pose several operational and environmental problems; namely, formation of potentially hazardous disinfection by-products (DBPs) and high energy consumption. Therefore, there is high demand for effective, low-cost disinfection treatments. Among advanced oxidation processes, the photocatalytic process, a form of green technology, is becoming increasingly attractive. A systematic review was carried out on the synthesis, characterization, toxicity, and antimicrobial performance of innovative engineered photocatalysts. In recent decades, various engineered photocatalysts have been developed to overcome the limits of conventional photocatalysts using different synthesis methods, and these are discussed together with the main parameters influencing the process behaviors. The potential environmental risks of engineered photocatalysts are also addressed, considering the toxicity effects presented in the literature.

## 1. Introduction

Waterborne pathogens, such as viruses, bacteria, and protozoa, are responsible for 3.5 billion cases of diarrhea each year and 1.8 million deaths. Most of them are children under 5 years of age [1,2]. Access to clean water and sanitation, listed as Goal 6 of the United Nation’s Sustainable Development Goals (SDGs), is one of the main challenges of the 2030 Agenda. Proper wastewater disinfection represents the base for the prevention of waterborne infections induced by pathogenic microbes, which can outbreaks and increase the disease burden, especially in developing countries, with subsequent social and economic impacts. Moreover, treated wastewater is also a reliable and attractive alternative source of water supply in developed countries (EU 2022/952 regulation). In this regard, disinfection is mandatory before wastewater discharge and reuse to kill/inactivate pathogens [3]. Contemporary conventional water disinfection technologies, including chlorination and UV, have been extensively discussed due to their various operational and environmental burdens, such as the formation of potentially hazardous disinfection by-products (DBPs), which are inevitably produced due to the reaction between disinfectants, halides, and organic matter, and high energy consumption. Previous studies have shown that the survival of various kinds of aquatic organisms, such as algae, cladocerans, polychaetes, and fish, may be threatened in water bodies receiving chlorinated WWTP effluent [3,4,5,6]. To date, approximately 800 DBPs have been discovered [7], and most of them are classified as genotoxic, carcinogenic, and cytotoxic [8]. Moreover, there are various concerns related to residual chlorine. After 96 h of exposure, just 14–29 μg/L of residual chlorine could kill 50% of rainbow trout [9].

Thus, the identification of alternative technologies is a necessary challenge for human health and ecosystem protection. A variety of treatment technologies have been reported in the literature to prevent the release of fecal indicator bacteria (FIB) (i.e., *Escherichia coli* and enterococci) into the environment via effluent discharge/reuse, with varying levels of success. Among them, advanced oxidation processes (AOPs) have emerged as promising, innovative water treatment technologies [10]. In particular, photocatalysis has gained growing attention owing to its intelligent design and adjustable operational efficiency and costs [2,11,12,13]. Titanium dioxide is the most common bulk and nanostructured semiconductor investigated for photocatalytic water disinfection and functions by inducing a series of redox reactions on its surface, activated by UV light. Under UV light, some of the photo-generated holes and electrons react with the H_2_O and O_2_ molecules adsorbed on its surface to initiate the formation of highly reactive oxygen species (ROS), which have an antibacterial effect through a mechanism involving oxidative stress, the hydroxyl radical being the major factor responsible for bacteria inactivation [14]. However, TiO_2_ has several drawbacks that limit its application, especially in large-scale industry, which include: (i) recombination phenomena affecting the free carriers, which are generated by the light irradiation; (ii) the value of the band gap (approximately 3.2 and 3.0 eV for anatase and rutile crystalline polymorphs, respectively [15]), which allows absorption only in the ultraviolet range (λ < 387 nm); (iii) the difficult separation of photocatalyst particles from the effluent. This has resulted in attempts to optimize process behaviors in several engineered photocatalysts designed recent decades, including: (i) immobilization of photocatalysts on a macroscopic support, eliminating the necessity of a separation process after water treatment and attenuating the problems concerning the ecotoxicity [16]; (ii) doped nanomaterials to reduce TiO_2_ band-gap energy, which enables it to absorb light in the visible region thanks to the formation of new energy levels among the conduction and valence bands of TiO_2_; and (iii) the creation of heterostructures (H) by combining different semiconductors, which helps in the separation of photogenerated charges by delocalizing them into different carriers and, thus, preventing them from recombining with each other [17]. Photocatalytic activity is greatly influenced by the physical structure and composition of the photocatalysts, and the costs and environmental sustainability of their preparation strongly affect full scale applications [17].

This review focuses on the synthesis, characterization, toxicity, and antimicrobial performance of engineered photocatalysts. Future perspectives for related research trends are summarized.

## 2. Methodology

### 2.1. Selection Protocol and Search Strategy

The present review was conducted according to the Preferred Reporting Items for Systematic Review and Meta-Analysis (PRISMA) guidelines in order to identify eligible articles to explore the antimicrobial effectiveness of innovative photocatalysts. The protocol was registered in PROSPERO (reference number CRD42021230625). The search was carried out using the databases PubMed, Science Direct, and Web of Science with the following terms: Innovative AND Photocatalyst* AND Disinfection. Title, abstract, and MeSH terms were used for the search in PubMed; the search in Science Direct and Web of Science included the topic according to the title, abstract, and keywords. The period from 1 January 2010 to 21 January 2022 was considered for the article collection.

### 2.2. Inclusion and Exclusion Criteria

Only observational studies and experimental and quasi-experimental studies written in English were considered eligible. Clinical trials, reviews, meta-analysis, case studies, proceedings, qualitative studies, editorials, commentary studies, and any other type of study were excluded from the database.

**Figure 1:**
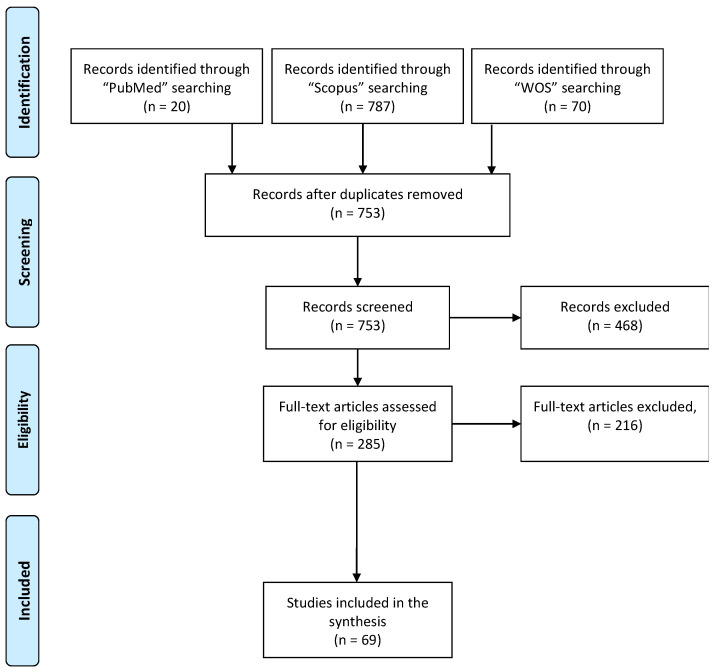
PRISMA flow diagram of the systematic review process.

Titles and abstracts acquired from the three databases were transferred to the reference site Covidence—Better Systematic Review Management [18] for the relevance assessment process. All the authors evaluated potentially eligible studies by title and abstract to establish if they met the inclusion criteria. The authors successively discussed their inclusion in the review. Agreement was achieved by consensus among the authors. The review process is represented in Figure 1.

## 3. Preparation and Characterization of Photocatalysts

All processes regarding the formulation of efficient photocatalysts require the study of synthesis procedures and physico-chemical characterization tests, which are useful to understand the mechanism by which a photocatalyst can work under light irradiation.

According to the literature, several methods can be used to prepare engineered photocatalysts, including sol–gel, hydrothermal-based, microemulsion, and precipitation methods, as well as their combinations [19,20]. The doping of photocatalysts makes it possible to inhibit the charge-carrier recombination phenomena and enables visible light absorption. The valance band holes, or conduction band electrons, are trapped in the defect sites generated by the dopant element, inhibiting the recombination of photo-induced holes and electrons and improving the interfacial charge transfer. Promising innovative catalysts have been produced using heterostructures. Heterojunctions can have synergic effects in various oxides, such as TiO_2_, SnO_2_, SiO_2_, CeO_2_, ZnO, WO_3_ and ZrO_2_, due to the injection of conduction band electrons, which decreases the recombination rate and increases electron–hole pair lifetimes.

This section describes the easiest and most cost-effective preparation methods for the synthesis of various doped or heterostructured semiconductors.

### 3.1. Sol–Gel Method

The sol–gel method is the most widely used method for the preparation of doped and undoped photocatalysts, and it can also be used in the nanometric range.

**Figure 2:**
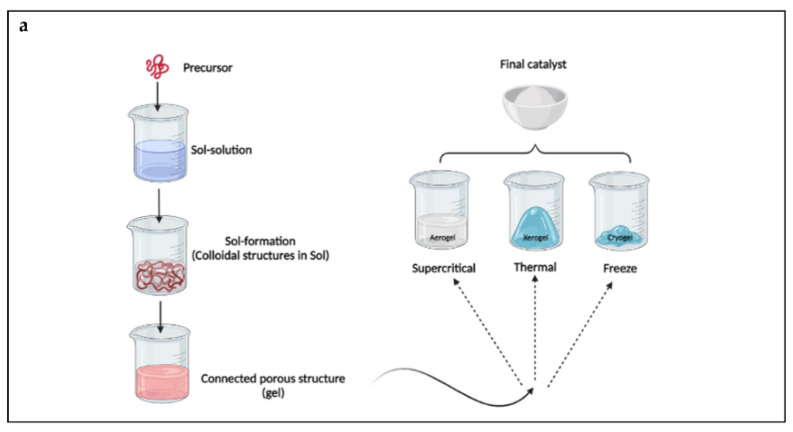

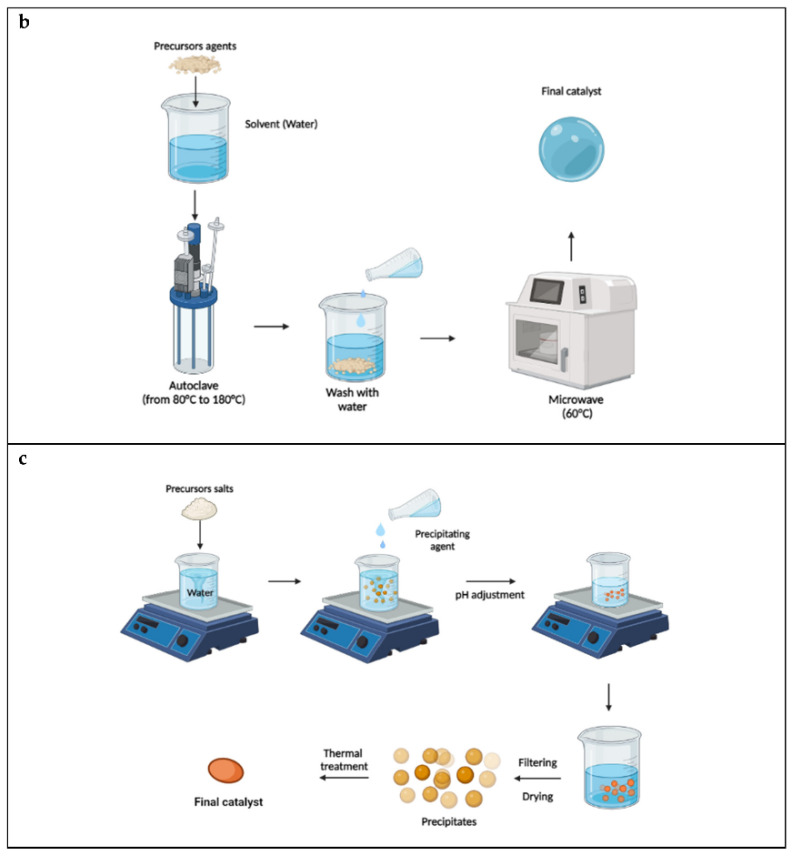
Schematic of different stages of (**a**) sol–gel process; (**b**) hydrothermal process; (**c**) precipitation method.

During sol–gel synthesis, the sol is generated by the hydrolysis and then polymerization of the precursor salt (usually metal alkoxides). The polycondensation reactions and the evaporation of the solvent make it possible to induce the transition from the sol to the gel phase. The process consists of the following steps: hydrolysis and condensation, then drying and thermal decomposition of precursors [17]. Figure 2a shows a schematic picture of a sol–gel process for the preparation of photocatalysts [21]. Depending on the solvent used, the sol–gel process can be classified as aqueous or non-aqueous.

As shown in Figure 2a, a molecular precursor is dissolved in water or alcohol and converted to gel through hydrolysis/alcoholysis using heating and stirring. The gel is wet or damp; thus, it needs to be dried using appropriate methods, depending on the desired properties and application. The sol–gel method makes it possible to obtain homogeneous composites with very high purity and is applicable at an industrial scale [22]. It is possible to create thin films with a thickness of 50–500 nm or powders. Different coating methods can be used to create thin film, including dip-coating, spin-coating, spray-coating, flow-coating, capillary-coating and climbing-cover processes [23,24,25].

Several doped photocatalysts have been prepared with the sol–gel process, including TiO_2_ doped with nitrogen and boron [26], nickel and cerium [27], fluorine [28], iron and zinc [29], and molybdenum and chromium [30]. ZnO has also been doped with various metals and non-metals, such as nitrogen, aluminum, silver, copper, and cobalt [31,32,33,34,35].

As a general remark, the steps in the sol–gel method can be changed to simplify the procedure and enhance the doping efficiency. In addition, sol–gel synthesis has also been adopted together with a dip-coating procedure to immobilize visible active photocatalysts on macroscopic and transparent supports, with the purpose of formulating structured photocatalysts for use in heterogeneous photocatalysis for the depollution of gaseous streams and in continuous fixed-bed photoreactors for wastewater treatment [36].

### 3.2. Hydrothermal Synthesis

Hydrothermal synthesis occurs in a closed vessel with controlled temperature and pressure. The temperature and pressure conditions facilitate the dissolution of the chemical reagents and the formation of the products through crystallization. This technique provides a one-step reaction route for the production of complex materials. The method is called “solvothermal” when a solvent other than water is used [37]. The synthesis of photocatalysts with this method is typically performed in steel vessels operating at high pressure (autoclaves) under controlled temperature, and the formation reaction of the nano-catalysts occurs in the liquid medium. A schematic picture of this method is shown in Figure 2b.

When the reaction mixture inside the autoclave is heated, two zones with different temperatures are created. The reactants of the mixture form a solution in the zone at higher temperature, while the saturated solution present in the lower part of the autoclave is transported to the upper section of the system due to convective motion. When the solution in the upper part of the autoclave becomes cooler and denser, it descends. Simultaneously, due to the temperature decrease, the solution exceeds the limit of solubility and precipitation begins. This technique makes it possible to directly obtain catalysts in powder form, and the crystalline degree can be tuned depending on the operating conditions. In addition, the particle size, shape, and chemical composition can be modified by changing only two parameters, the temperature of the reaction mixture and the solvent used in the synthesis, in order to reach a high pressure and, consequently, supersaturation at lower temperatures.

When this method is used for the preparation of photocatalysts, it has been shown to be very effective in incorporating dopants into the crystalline structure of TiO_2_ and ZnO. Many studies have been devoted to the controlled synthesis of TiO_2_ particles in particular due to their high photocatalytic activity [38].

F-doped, hollow TiO_2_ microspheres were prepared by Zhou et al. [39] through a hydrothermal synthesis method, controlling the hydrolysis of TiF_4_ in an autoclave made of Teflon at a reaction temperature of 180 °C.

A visible, active N-doped TiO_2_ photocatalyst was prepared using triethylamine as a nitrogen source with a low-temperature hydrothermal method [40].

Hydrothermal methods have also been used to prepare photocatalysts other than TiO_2_ and ZnO, forming structures with very high degrees of crystallinity. For example, Amano et al. [41] showed that bismuth tungstate (Bi_2_WO_6_) prepared following hydrothermal synthesis possessed high photocatalytic efficiency under visible light irradiation.

The increasing interest in hydrothermal synthesis derives from its advantages, such as the high reactivity of the reactants, easy control over the solution or interface reactions, the formation of metastable and unique condensed phases, less air pollution, and low energy consumption. The nanostructured energy materials can grow directly on conductive substrates with good, solid contact that can strongly enhance the conductivity [41].

### 3.3. Precipitation Method

The preparation of photocatalysts through the precipitation method consists of the chemical transformation of a highly soluble metal precursor salt into a chemical compound with lower solubility (Figure 2c).

The generation of the weakly soluble compound (and then the precipitate) is usually undertaken by changing (generally by increasing) the solution pH [42,43].

The semiconductor most widely prepared with this method is ZnO. Generally, the precursor of ZnO is obtained using a direct precipitation method involving the reaction between a zinc salt and a base in an aqueous solution, which belongs to the solution phase [44]. In particular, the preparation involves the reaction of zinc salts, such as Zn(NO_3_)_2_, Zn(CH_3_COO)_2_·2H_2_O, ZnSO_4_, etc., with a basic solution containing, for example, NH_4_OH or NaOH [45].

To dope ZnO with metals (with the aim of shifting its absorption in the visible region), the precursor salt of the doping element can be added to the solution of the zinc precursor before inducing the precipitation with the basic solution [46,47]. The obtained precipitate is then transformed into doped ZnO photocatalysts through thermal treatment.

### 3.4. Microemulsion

Microemulsion is a preparation process with which it is possible to control the morphological and structural parameters of both semiconductor particles and heterostructures [48]. In detail, direct (oil-in-water) and inverse (water-in-oil) microemulsion media can be used to prepare different photocatalysts. Microemulsions are thermodynamically stable solutions containing, at the least, a polar phase (usually water), a nonpolar phase (usually oil), and a surfactant. Different microstructures can be generated, ranging from droplets of oil dispersed in a water phase (oil-in-water) over a bi-continuous “sponge” phase to water droplets dispersed in a continuous oil phase (water-in-oil) [49]. The latter can be used as nanoreactors for the preparation of nanoparticles [50]. In the case of photocatalytic materials, the first step in nanoparticle formation is the chemical reaction between the two reactants trapped in the microemulsion cores, or the reaction between the reactant and the precipitating agent. For instance, TiO_2_-based photocatalysts can be prepared through the direct reaction of titanium isopropoxide with water solubilized in water-in-oil microemulsions stabilized by the presence of a surfactant, such as Triton X-100 [51]. For ZnO-based materials, zinc nitrate has been solubilized in the aqueous phase of the microemulsion together with a precipitation agent (such as tetramethylammonium hydroxide pentahydrate) [52]. However, in most cases, a final thermal treatment is required to obtain the desired crystalline phase for the semiconductor particles.

### 3.5. Characterization of Photocatalysts

It is very important to collect information on the physico-chemical properties of engineered photocatalysts in order to understand the effect of the operating parameters adopted in the synthesis procedure. A wide variety of characterization methods are available, which are discussed in the literature; thus, they are only briefly described here. 

Electron microscopy (SEM, TEM), atomic force microscopy (AFM), Raman spectroscopy, X-ray diffraction (XRD), and X-ray photoelectron spectroscopy (XPS) are typically used to characterize photocatalyst structure.

To determine the photocatalyst morphology at very high magnifications, scanning electron microscopy (SEM) is used. SEM analysis makes it possible to collect information about agglomerate size and shape. Transmission electron microscopy (TEM) permits higher magnifications than SEM.

To define the distribution and specific surface area (SSA) of pores, N_2_ adsorption–desorption measurements at −196 °C are required. This analytical technique is based on the physical adsorption of gaseous molecules on the catalyst surface and within its pores. Since all the semiconductors used in photocatalysis are typically mesoporous materials, the most widely used model to measure SSA is the Brauner–Emmett–Teller (BET) model. Generally, a greater surface area is linked to an increase in photocatalytic activity [53]. The BET method is, however, extensively discussed in the literature [54].

X-ray diffraction analysis (XRD) is commonly used to identify the crystalline phase of photocatalysts through Bragg’s law [55]. Additionally, the Sherrer equation makes it possible to estimate the crystallite size of photocatalysts [56]. 

Another useful method is Raman spectroscopy [57], which is based on the measurement of the Raman shift. The resulting plot displays the intensity as a function of the Raman shift. The use of Raman spectroscopy for the characterization of semiconductor photocatalysts makes it possible to highlight possible contaminants on the surface of the engineered photocatalyst (such as metal or non-metal groups bonded only on the external catalyst surface) and correlate them with photocatalytic activity. Contaminants on the surface can act as recombination centers for electron/hole scavengers, inducing a worsening of the photocatalytic activity.

The most important traditional technique used for the analysis of photocatalysts is UV-visible diffuse reflectance spectroscopy (UV-Vis DRS). UV-Vis DRS can analyze the light absorption properties of different materials. UV-Vis diffuse reflectance spectrophotometers provide data that are useful for the estimation of the band gap in semiconductors [58]. To this purpose, mathematical elaborations (e.g., Tauc plots) can be used to estimate the band-gap energy. 

Recently, more refined techniques have been used for photocatalyst characterization. For instance, electron paramagnetic resonance (EPR) can be used to check the possible formation of reactive oxygen species under irradiation. Additionally, EPR is extremely powerful for understanding the nature of photoactive defects [59]. Together with EPR analysis, density functional theory (DFT) calculations can give detailed information about the change in electronic structure induced by the doping of semiconductors in order to understand the effect of the interaction of semiconductors with a specified light source [60].

Finally, time-resolved photo-luminescence (TRPL) can also be used to assess the evolution of the photocatalyst luminescence spectrum as a function of time [61], making it possible to analyze the charge-carrier lifetime and dynamics within a particular system.

## 4. Antimicrobial Efficiencies

Antimicrobial efficiencies depend on numerous factors, including the type and dose of catalyst, the type of microbe, the intensity of radiation, the degree of hydroxylation, the pH, the temperature, and the exposure time. Data for different applications of photocatalysts are reported in Table 1, Table 2 and Table 3. Antimicrobial efficiency effects comprise a wide range of endpoints that can be estimated on a study-by-study basis, such as bactericidal, bacteriostatic, and antiviral effects. The most common approach is to verify the decrease in the initial microbial load after the treatment as a percentage of reduction.

Most engineered photocatalysts are designed to be active under visible light when not directly under solar light, and only a few cases use UV [16,68,89,101] or actinic light (max wavelength at 420 nm) [95,100]. To improve visible photocatalytic activity and to minimize the recombination phenomena in the generated electron-holes, several modifications of TiO_2_ using metals, non-metals, cations, and anions have been attempted [1,62,63,64,66,72,76,85,88], creating heterostructures (Table 1), doped photocatalysts (Table 2) and polymer nanocomposites (Table 3). An emerging field of interest is the synthesis of TiO_2_ nanotubes and their coupling with cations, metal-oxides, and additional composites, leading to a higher sensitization in the visible range [70,85].

The use of Zn to improve the ability of TiO_2_ to work under solar and UV light has been widely exploited in heterostructures and doped materials (Table 1 and Table 2). As reported in Table 2, Stoyanova et al. [106] observed that, after 20 min of photocatalytic treatment, 1 g/L of TiO_2_/ZnO, made it possible to achieve 100% removal of 10^5^ UFC/mL *E. coli* in the presence of UV light. Similar results were reported by Sethi et al. [1] (Table 2) after only 10 min of treatment using visible radiation with the lowest power compared to all other selected experimental studies. Wang et al. [107] reported that ZnCl_2_/TiO_2_ and Zn(Ac)_2_/TiO_2_ nanoparticles were more efficient in the removal of *C. albicans* than *E. coli* and *S. aureus* using visible light (Table 2).

Due to its high stability, abundance, and matching band position with TiO_2_, Fe_2_O_3_ is one of the surface co-catalysts used to create heterostructures for the control of electron-hole pair recombination in semiconductor-based photocatalysts [83]. TiO_2_–Fe_2_O_3_ nanocomposites proved to be an efficient photocatalyst in terms of the inactivation of *E.coli* (99% removal) under direct natural sunlight irradiation but required a significantly greater treatment time (up to 120 min) [80] than under UV light (30 min) [68].

Furthermore, several other visible light-driven (VLD) photocatalysts, including Bi-based [73,81,96], Ag-based [64,66,67], and C-based photocatalysts, have been recently investigated. In particular, bismuth oxyhalide (BiOI), a p-type semiconductor, showed the strongest visible light absorption due to its narrow band gap (1.7–1.8 eV) [66]. Heterostructures created by coupling TiO_2_ with BiOI make it possible to achieve improved visible light catalytic behaviors. However, the mismatch in the band alignment between TiO_2_ and BiOI limits the interfacial charge transfer. To overcome this limit, a recent study co-decorated TiO_2_/BiOI nanoparticles with Ag nanoparticles to a more efficient photocatalysts with broad light absorption and efficient charge transfer [66]. As reported in Table 1, the complete removal of 3 × 10^7^ UFC/mL *E. coli* could be achieved after 30 min with a 16 W visible light lamp. 

Molybdenum disulfide (MoS_2_), a p-type semiconductor, has also been exploited as a co-catalyst to expand the response range of TiO_2_ to visible light and improve the efficiency of photogenerated charge separation. MoS_2_/TiO_2_ nanotube arrays prepared by coupling MoS_2_ with the n-type semiconductor TiO_2_ determined the formation of a p-n heterojunction between MoS_2_ and TiO_2_, making it possible to achieve a sterilization effect per unit area of MoS_2_ nanotubes close to that of some powder dosage photocatalysts under visible light irradiation [70] (Table 2).

Graphitic carbon nitride (g-C_3_N_4_) has emerged as an innovative visible-light photocatalyst for environmental applications [74,75]. g-C_3_N_4_ modified with AgBr [71], V-TiO_2_ [62], expanded perlite [74], and graphene [86] has shown strong antibacterial capacity against *E. coli* cells with visible light (Table 1), but no applications have been reported with sunlight. Recently, 2D engineered photocatalysts and their composites [110], including Ag- and graphene oxide (GO)-based composites, have gained much attention due to their effective antimicrobial activity. It was been demonstrated that the interaction between GO and plasmid DNA inhibits the amplification and transformation of *aphA* genes. Moreover, the inhibition increases with the decreasing size of the GO [111,112]. The heterostructures created by combining g-C_3_N_4_ with graphene oxide (GO/g-C_3_N_4_) could kill 97.9% of *E. coli* after 120 min visible light irradiation at the concentration of 100 μg/mL (Table 1). It has been observed that Ag nanoparticles constitute an effective interfacial bridge between binary semiconductor nanocomposites. To date, various Ag-modified ternary photocatalysts, such as Ag/QDs/BiS_3_/SnIn_4_S_8_ [69], AgI/AgBr/BiOBr_0.75_I_0.25n_ [73], and Ag-AgX/RGOs [78], have been developed, exhibiting improved photocatalytic performance under visible light irradiation, which is mainly related to the surface plasmon resonance (SPR) and Schottky effect of metallic Ag nanoparticles. Among these studies, the photocatalytic process proposed by Liang et al. [73] achieved the best results, with the lowest concentration of photocatalysts and the highest concentration of *E. coli* (3 × 10^7^ UFC/mL), using a 300 W visible light lamp.

### 4.1. Photocatalyst Dose

There is no agreement in the literature regarding the influence of photocatalyst dose on process behavior, as it strictly depends on the form (particles, nanoparticles, film) and specific characteristics of the photocatalysts. According to Li et al. [67], the dosage of catalyst influenced the photocatalytic disinfection efficiency. An increase of the inactivation level for viruses from ~4.5 log to ~6 log was observed when increasing the photocatalyst concentration from 50 mg/L to 100 mg/L; a maximum value of ~8 log was achieved at a photocatalyst concentration of 150 mg/L after 360 min visible light illumination. On the other hand, by increasing the g-C_3_N_4_ concentration up to 200 mg/L, a decrease in virus MS2 inactivation to 7.5 log could be observed. This result was predictable since the addition of a large amount of photocatalysts can lead to a great decrease in light penetration. Thus, an optimum dosage for photocatalysts is critical for process optimization.

### 4.2. Effect of pH

In tests of the photocatalytic disinfection activity of different photocatalysts towards pathogenic bacteria under various pH conditions, the cell density did not decrease significantly under neutral–acidic pH [71,81]. The antibacterial efficiencies of g-C_3_N_4_-AgBr were similar under neutral and slightly acidic conditions of pH 5–7. The acidic condition resulted in the release of Ag^+^; however, its contribution to cell disinfection was estimated to be negligible due to the low concentration [71]. On the other hand, alkaline conditions enhanced the disinfection activities of g-C_3_N_4_-AgBr, making it possible to achieve the best performances at pH 8 and pH 9. The increasing solution pH did not induce change in the zeta potentials of g-C_3_N_4_-AgBr, while the zeta potentials of *E. coli* became slightly more negative at high pH. As expected, the electrostatic force between bacteria and g-C_3_N_4_-AgBr was more repulsive under alkaline conditions [71].

According to Zhang et al. [74], faster viral inactivation by g-C_3_N_4_/EP-520 could be observed after decreasing pH values. At the same reaction time, about 5 log of inactivation was observed with 180 min visible light irradiation at pH 9, while 8 log of inactivation was achieved at pH 5. Reduced electrostatic repulsion between MS2 and g-C_3_N_4_ produced by the acidic pH was considered responsible for the change in viral inactivation. MS2 has an isoelectric point of 3.9 and was negatively charged at all pH levels. g-C_3_N_4_ shows an isoelectric point of 5.0, and its overall negative charge decreased as pH decreased from 9 to 5, facilitating MS2/g-C_3_N_4_ interaction.

### 4.3. Effect of Temperature

Few studies have investigated the effects of temperature. However, it is well-known that, by increasing the temperature, photocatalytic reaction activity is enhanced. Accordingly, Basu et al. [2] reported that, with the increase in reaction temperature, bacterial disinfection time decreased. Nevertheless, a detailed explanation of how temperature affects photocatalytic inactivation needs to be provided [113].

### 4.4. Target

Most studies to date have focused on the fecal indicator bacteria (FIB) *E. Coli*. Only a few have investigated viral inactivation with visible light-active photocatalysts in water [74] (Table 1), and one study investigated the effect on fungi inactivation [91]. For instance, bacteriophage MS2, a widely used surrogate for waterborne pathogenic viruses due to their similar size, structure, and surface properties, was selected as a model virus in the study by Zhang et al. [74]. Viruses and fungi are more resistant than bacteria to conventional disinfection methods [111], and the results of bacterial disinfection cannot be translated to viral disinfection. The mechanism of the photocatalytic inactivation of viruses is still largely unknown [67]. Considering that real water systems usually contain consortia of different bacteria (e.g., Gram-positive and Gram-negative), it would be highly recommended to investigate photocatalysts’ efficiency against other bacterial systems to achieve a complete evaluation of these processes.

### 4.5. Effect of Water Matrix

Zhang et al. [74] investigated the effect of the water matrix on disinfection during photocatalytic inactivation of MS2, reporting that the viral inactivation efficiency in real source water was lower than that in deionized water with 240 min visible light irradiation (3.7 vs. 8 log removal). The main reason for the reduced disinfection efficiency can be ascribed to the presence of natural water constituents; e.g., natural organic matter, which can be adsorbed on photocatalysts to prevent ROS generation or to consume generated ROS, acting as scavengers.

### 4.6. Role of Direct Contact

Process behavior is strongly affected by the direct contact between photocatalysts and bacterial cells. However, long-range disinfection activity that did not depend on direct contact has also been reported previously [81].

Microbial inactivation can be achieved by photocatalysis-mediated reactive oxygen species (ROS), which work in the cell wall. The ROS in intimate contact with bacteria induce the peroxidation of the polyunsaturated phospholipid component of the lipid membrane and promote the disruption of cell respiration to destroy bacteria [63]. However, microorganisms with a more complex cell wall structure, such as Gram-positive bacteria, are likely more resistant to ROS.

### 4.7. Influence of Light

The power of the lamps used in the selected experimental studies varied from 8 W [1] to 500 W [62]. However, a higher-power light source did not correspond to better process behavior, as it represents only one of the variables influencing the microorganism inactivation. 

## 5. Discussion

### 5.1. Synthesis Methods

All the preparation methods described in Section 3 require the use of solvents and/or corrosive chemicals. For this reason, despite these methods producing engineered photocatalysts with high activities, special attention should be paid to green and environmentally friendly synthesis in order to minimize the possible negative impact on the environment due to the low sustainability involved [114]. General advantages of chemical methods are easy surface functionalization and versatility if nanomorphology formation, which make it possible to enhance their potential uses in different environments.

Among the various sustainable and green synthesis routes, electrochemical methods show the following advantages: (i) use of chemical agents commonly employed in wet-chemical synthesis routes [115]; (ii) the crystal growth rate of particles can be easily tuned using deposition potentials, current densities, or salt concentrations [116]; (iii) doping elements, such as Cu, can be easily introduced into the semiconductor lattice [117].

**Figure 3:**
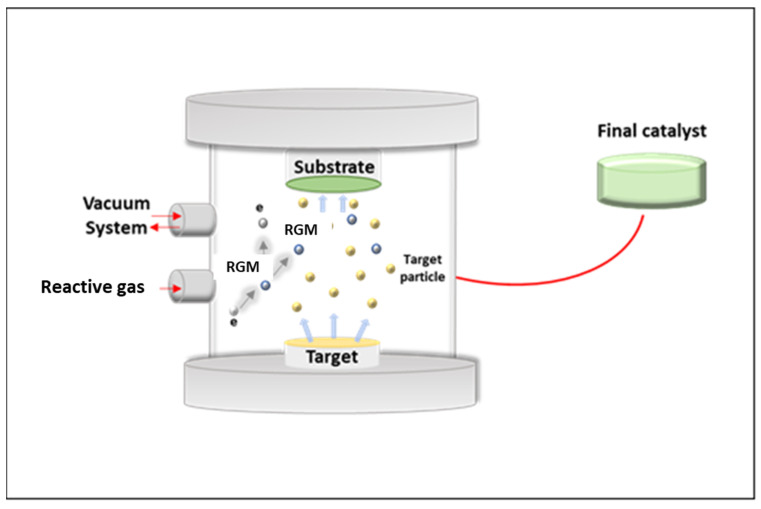
Schematic representation of the sputtering process. RGM: reactive gaseous molecule.

In the field of electrochemical methods, a very interesting green preparation method could be based on sputtering techniques, which, moreover, offer the possibility of developing photocatalysts immobilized on a macroscopic support, thus avoiding the need to separate powder photocatalysts from the treated water [118]. Generally speaking, the sputtering method presents several advantages, such as coating uniformity over large areas, good control of morphological properties in the photocatalytic films, and lack of toxic or hazardous precursors [119]. Additionally, it has been extensively reported that the sputtering method is able to produce photocatalytic films that have higher durability compared to sol–gel techniques [120]. Moreover, reactive gases, such as oxygen or air, can be introduced into the process to react with the sputtered metal atoms, resulting in the formation of a photocatalytic film (Figure 3) [121].

As is possible to observe from Table 1, several photocatalysts have been prepared using this method.

Alternative green approaches are based on mechanochemistry methods [121], such as the milling route [122]. This method is based on the use of a milling vessel loaded with the milling media (such as balls) and reactants [123]. In some cases, additional chemicals are added to the milling mixture with the aim of minimizing particle agglomeration. Finally, the milled material is recovered after a certain treatment time with a certain milling frequency [123]. The milling route can be used for the synthesis of heterostructures as an alternative to hydrothermal or solvothermal methods.

### 5.2. Regrowth

Regrowth tests are necessary to provide further insight into the effect of disinfection processes on microorganisms’ inactivation. None of the selected experimental studies performed regrowth tests, which would be required to verify the total inactivation of target microorganisms in the photocatalysis process instead of simply suppressing their growth and reproduction abilities.

### 5.3. Reusability of Photocatalysts

The reusability and stability of photocatalysts play significant roles in practical applications of disinfection. Feng et al. [81] reported that the bactericidal efficiencies of BiOBr-0.5AgBr were slightly decreased with the increase in reuse cycles. According to the studies by Shanmugam et al. [62], the g-C_3_N_4_-10% V-TiO_2_ hybrid photocatalyst still showed outstanding photocatalytic stability after up to five cycles of reuse. Shi et al. [65] performed recycle experiments with CuBi_2_O_4_/Bi_2_MoO_6_, observing that the FT-IR and XRD analyses displayed almost no change in the crystal phase and transmission peaks over time, demonstrating that the photocatalyst still preserved high photocatalytic bactericidal activity towards *E. coli*. A decrease in the inactivation property was attributed to the loss of the photocatalyst during the recovery process.

### 5.4. Toxicity Evaluation

Traditional animal models and assays have been historically applied to determine the potential human and ecological hazards and risks of compounds through the evaluation of various endpoints (i.e., embryo lethality, reproductive and developmental toxicity, genotoxicity, carcinogenicity, neurotoxicity, etc.) [124,125,126,127,128,129]. As reported in Table 4, few studies have focused on the ecotoxicity of engineered photocatalysts so far, probably due to the scarce availability of standardized protocols. Moreover, they concurred with antimicrobial applications only in a few cases.

Chen et al. [130] explored the aquatic toxicity of water treated with silver phosphate (Ag_3_PO_4_) photocatalyst against *Chlorella vulgaris*, observing a greater stimulatory effect on the growth of algae with respect to the control (algae exposed to untreated water). ZnO@ZnS-based photocatalysts displayed negligible effects on the viability, biomass, and photosynthetic pigments of *Spirulina platensis* microalgae [131]. Similarly, nitrogen-doped TiO_2_ showed a reduction in toxicity in terms of *Vibrio fischeri* and *Raphidocelis subcapitata* growth and *Daphnia magna* survival after 300 min of wastewater (contaminated with various pharmaceuticals) treatment [132].

The toxicity of hydrogen (H_2_RGOTi)- and thermal (RGOTi)-reduced graphene oxide/TiO_2_ has been investigated for zebrafish embryos, showing that H_2_RGOTi could be more ecofriendly than RGOTi [133] (see also Table 4). In fact, RGOTi was able to increase mortality (LC_50_ = 0.7 g/L; Table 4) and the size of the eye, yolk, and pericardium, with consequent cardiac development damage [134]. Instead, the facet-dependent monoclinic scheelite BiVO_4_ (m-BiVO_4_) weakly affected the survival and the development of zebrafish embryos [134]. Recently, biochar functionalized with titanium dioxide (TiO_2_) was evaluated for its effects on the survival, neurotoxicity, and energy metabolism of *Mytilus galloprovincialis* bivalves, showing effects comparable to those observed in the controls [135]. In 2021, an in vivo toxicity study of the effects of water treated with alumina/ZnO on female pathogen-free Balb/c mice revealed high bacteria disinfection and no impact on gut health [2]. In contrast, when exposing male Wistar rats to Fe_2_O_3_ nanoparticles, Abhilash et al. [136] demonstrated that heart tissue and, consequently, the cardiovascular system suffered toxic damage. In the same manner, the cerium oxide/sulfide nanoparticles in the zeolite channels displayed a toxic impact on the number of white blood cells and hemoglobin level of rats [137]. Various studies have also been conducted on mammal cell lines, showing negligible effects most of the time (see Table 4). El Nahrawy et al. [138] showed a negative effect toward skin cell lines in laryngeal carcinoma (Hep-2) after zinc titanate (Zn_2_TiO_4_) exposure. In the same manner, silver nanoparticle-modified titanium (Ti-nAg) did not affect human gingival fibroblasts [139], whereas Ag nanoparticles@chitosan–TiO_2_ showed low toxicity toward mammalian cell [109]. The TiO_2_:Cu nanocomposite showed beneficial effects on embryonic mouse fibroblast cells, with an enhancement of about 20% in cell viability [105]. Malankowska et al. [140] compared the sensitivity of two human cell lines (lung cells (A549) and liver cells (HepG2)) and one mouse cell line (embryo fibroblast cells (BALB/3T3)) to multicomponent (silver (Ag), gold (Au), platinum (Pt), and palladium (Pd)) TiO_2_-based photocatalysts, finding that the HepG2 and A549 cells were, respectively, the most and least sensitive among all the cell lines (see Table 4). Furthermore, oxygen-doped graphitic carbon nitride microspheres (O-g-C_3_N_4_) and hydrogen-doped zinc oxide (ZnO(H)) displayed negligible cytotoxicity towards A549 cells [141,147]. Fe/Cr-doped CeO_2_ NPs showed negative effects on the aneuploid immortal keratinocyte (HaCaT) cell line [142]. The potential cytotoxic effects of Fe-doped TiO_2_ on human endothelial cells (HECVs), red blood cells, hemocytes of *Mytilus galloprovincialis*, and mouse macrophages (RAW 247) were evaluated, showing a decrease in cell viability only for HECV [143,144,145].Cadmium–bismuth microspheres (CdS-Bi_2_S_3_) exhibited high cytotoxicity activity against a human colon colorectal tumor (HCT 116) cell line, even at the lowest tested concentration (0.25 g/L; see Table 4) [146].

The paucity of data concerning ecotoxicological implications reported in these few studies does not permit definitive estimates of the types and degrees of toxicity generated by the engineered photocatalysts when they are released into aquatic environments [148,149,150] and whether their interaction with biota can induce potentially adverse effects at different biological levels [151,152,153]. As a result, the toxicological risk applies not only to aquatic species but also to human beings, who could be exposed to such products through marine food webs [154,155]. Moreover, when applied to real wastewater, the process can generate dangerous intermediates from the degradation of organic contaminants. Thus, further studies are necessary to elucidate the ecotoxicity of effluents and of innovative photocatalyst nanoparticles themselves.

### 5.5. Photoreactor Configurations

**Figure 4:**
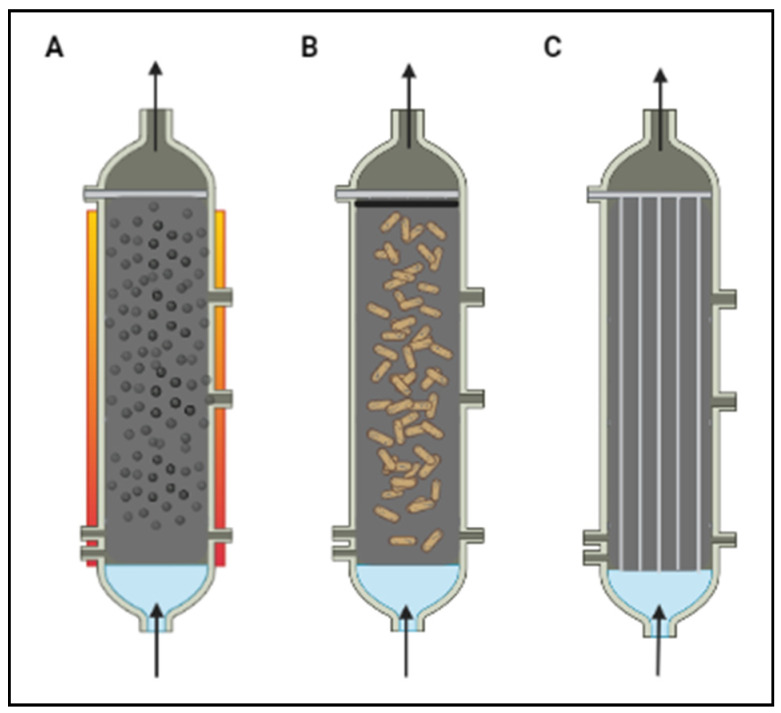
Photocatalysis reactor configuration: (**A**) free NPs separated from the water via a magnetic field; (**B**) NPs immobilized on macroscopic particles (fixed or fluidized bed); (**C**) NPs immobilized on membranes.

The photocatalysts used in powder form are characterized by a large surface area and are more uniformly mixed in the solution, showing excellent bactericidal effects (Table 1, Table 2 and Table 3). Nevertheless, photoreactors designed to use photocatalyst suspensions require high energy consumption and secondary filtration to separate the nanomaterials from water [156] (Figure 4).

The need to use an immobilized catalyst rather than catalyst powder in slurries has therefore been pointed out by recent studies. Indeed, different supporting materials, such as glass, ceramics, activated carbon, and polymeric materials, have been investigated [156,157,158,159,160]. 

Regarding polymers, the immobilization of the nanostructures in these types of materials not only makes it possible to avoid the separation step after water treatment but also reduces the problems concerning ecotoxicity and the aggregation of nanomaterials. This is the case, for instance, when poly (methyl methacrylate) (PMMA) is used as a polymer matrix in the preparation of nanocomposites (Table 3). PMMA, a common thermoplastic material, is used in many applications due to its transparency to visible light, mechanical properties, and environmental stability; it is also an economical and hydrophobic polymer suitable for contact with food and beverages. PMMA is an excellent host for functional inorganic particles; in fact, various types of metal oxide fillers have been demonstrated to further improve its properties [14].

Song et al. [65], faced the challenge of developing ternary, highly active TiO_2_-based photocatalysts with a novel structural form, good operability, and easy recyclability, created a flexible and hierarchical heterostructured Ag/BiOI/TiO_2_ nanofibrous membrane. 

### 5.6. Electric Energy Consumption

Unfortunately, fabrication of engineered photocatalysts is complicated and expensive, limiting their mass production and engineering applications. To evaluate the possibility of designing a photocatalytic system working under visible light, it is necessary to consider the treatment times, the ability to remove toxicity, and the energy consumption required for the treatment. Despite several engineered photocatalysts being able to exploit visible light, leading to complete removal of bacteria concentrations, only a few of them can achieve this result in a reasonable time. By considering a treatment time of 10 min, three photocatalysts were selected for the evaluation of electric energy consumption using the EE/O value—a scale-up parameter for removal of 90% of a pollutant contained in 1 m^3^ of polluted water, expressed in kWh in European countries—according to the following equation (Azbar et al. [161] and Vaiano et al. [162]):

$$E_{E/O}=\frac{P\cdot t\cdot 1000}{V\cdot 60\cdot \ln\left(\frac{C_{0}}{C_{f}}\right)}$$

where *P* is the nominal power of the light source (kW), *t* is the irradiation time (minutes), *V* is the volume solution (L), *C*_0_ is the *E. coli* initial concentration (UFC/mL), and *C_f_* is the *E. coli* final concentration. Assuming 10 min as the treatment time, we compared the EE/O values of two photocatalysts, ZnO/TiO_2_ [1] and Cu/ TiO_2_ [111].

The calculation of electric energy consumption showed that the use of ZnO/TiO_2_ [1] (EE/O = 0. 193 kWh/m^3^) under visible light used about 90% less energy compared to the use of Cu/TiO_2_ (EE/O = 2.11 kWh/m^3^) [111] under simulated sunlight. However, it must be considered that the electric energy consumption is strongly dependent on both the photoreactor configuration and photocatalyst composition. In fact, different results could be obtained by using lamps with the same power but different photocatalytic systems.

## 6. Conclusions

In the last decade, several studies have focused on the antimicrobial properties of engineered photocatalysts. Despite the promise of these materials, several issues related to their use still remain to be addressed:

- Toxicological and ecotoxicological aspects have not been fully investigated and should be carefully assessed before planning full-scale production;
- Greening production and minimizing the use of solvents should be considered essential for large-scale application;
- Pilot-scale plant experiments are necessary to carry out a realistic cost evaluation per unit volume;
- Regrowth and reuse have to be considered for a complete assessment of behaviors.

## Figures and Tables

**Table 1:** Heterostructured photocatalysts used for microorganism inactivation.

Photocatalyst	Form	Preparation Method	Dose (g/L)	Contact Time (min)	Target	UFC/mL	Light Source	Power of Light Source (W)	Results (%)	References
g-C_3_N_4_-V-TiO_2_	P	Hydrothermal calcination	0.5	60	*E. coli*	-	Vis	500	99.5	[62]
					*S. aureus*					
ZnO/TiO_2_	P	Wet impregnation calcination	0.5	10	*E. coli*	10^7^	UV Vis	8	99.9	[1]
Au/BiTiO_3_/TiO_2_	P	Hydrothermal treatment Ion exchange treatment Physical vapor deposition (PVD) process	-	40	*E. coli*	6 × 10^4^	Simulated sunlight	-	99.5	[63]
					*S. aureus*				99.7	
Ag_2_C_2_O_4_/TiO_2_	NF	Electrospinning Calcination	0.1	30	*E. coli*	2 × 10^7^	Vis	300	99.99	[64]
CuBi_2_O_4_/Bi_2_MoO_6_	P	Hydrothermal treatment Ultrasonication Heating	0.8	240	*E. coli*	10^7^	Vis	300	100	[65]
Ag/BiOI/TiO_2_	NF	Electrospinning ionic layer adsorption and reaction (SILAR) photodeposition	-	30	*E. coli*	3 × 10^7^	Vis	16	99.9	[66]
Ag_2_WO_4_/g-C_3_N_4_	P	Deposition Precipitation Ultrasonication	4	90	*E. coli*	10^7^	Vis	300	100	[67]
Fe_2_O_3_-TiO_2_	P	Ultrasonic co-precipitation	1.05	30	*V. fischeri*	3 × 10^6^	UV	-	100	[68]
Ag QDs/Bi_2_S_3_/SnIn_4_S_8_	P	Solvothermal method	-	240	*E. coli*	2.5 × 10^7^	Vis	300	100	[69]
MoS_2_/TiO_2_	NT	Two-step anodization Hydrothermal method	-	150	*Staphylococcus aureus*	>10^8^	Vis	-	100	[70]
					*E. coli*					
g-C_3_N_4_-AgBr	P	Adsorption–deposition	0.1	150	*S. aureus*	3 × 10^6^	Vis	300	100	[71]
				60	*E. coli*					
TiO_2_-rGO	P	Hydrothermal method	0.1	75	*E. coli*	1.5 × 10^6^	Artificial solar light	-	100	[72]
AgI/AgBr/BiOBr_0_․_75_I_0_․_25n_	P	Solvothermal method	0.08	30	*E. coli*	3 × 10^7^	Vis	300	100	[73]
g-C_3_N_4_/expanded perlite (EP-520)	P	Thermal method	-	120	*E. coli*	1 × 10^8^	Vis	300	100	[74,75]
				240	*MS2*					
Al_2_O_3_/ZnO	P	Co-precipitation Calcination	0.5	240	*E. coli*	10^6^	Vis	-	100	[2]
TGP (TiO_2_–graphene sensitized by tetrakis(4-carboxyphenyl)porphyrin (TCPP))	P	Solvothermal method	-	440	*E. coli*	-	Vis	450	64	[76]
AgI@MnO_2_	P	Deposition	0.05	25	*S. aureus*	-	Vis	15	99.4	[77]
					*E. coli*				92.2	
Ag-AgX/RGOs	S	Deposition Precipitation	-	35 min	*E. coli*	2 × 10^7^	Vis	300	100	[78]
CeO_2_-AgI,	P	Hydrothermal method Deposition	0.1	40	*E. coli*	10^7^	Vis	-	100	[79]
O-g-C_3_N_4_/HTCC-2	MS	Solvothermal method Hydrothermal method	0.15	120	*Viruses*	10^5^ MPN/mL	Vis	-	100	[80]
BiOBr-AgBr	P	Precipitation Ion exchange	0.08	24	*E. coli*	1 × 10^7^	Vis	-	100	[81]
BiVO_4_/Ag^+^	P	Hydrothermal method	0.1	15	*E. coli*	10^8^	Vis	-	>99	[82]
TiO_2_–Fe_2_O_3_	P	Ex situ synthetic route Ultrasonication	-	120	*E. coli*	3.22 × 10^9^	Sunlight	-	98.3	[83]
TiO_2_-X/Ag_3_PO_4_	P	Hydrothermal method	0.2	20	*S. aureus*	10^7^	Simulated sunlight	-	99.8	[84]
					*E. coli*				99.8	
Ag_(3%)_-TiO_2_	NT	Hydrothermal method	0.1	60	*E. coli*	10^6^	Sunlight	-	100	[85]
GO/g-C_3_N_4_	P	Sonochemical method	0.1	120	*E. coli*	10^7^	Vis	-	100	[86]

P: particles, F: film, NF: nanofiber, PF: polymer functionalized, NT: nanotube, NTAs: nanotube arrays, S: sheet, MS: microsphere, NS: nanosheet.

**Table 2:** Doped photocatalysts used for microorganism inactivation.

Photocatalyst	Form	Preparation Method	Dose (g/L)	Contact Time (min)	Target	UFC/mL	Light Source	Power of Light Source (W)	Results (%)	References
TiON	F	Sputtering on polyester (4 min)	-	40	*E. coli*	10^6^	Simulated sunlight	128	100	[87]
TiO_2_-Cu	F	Sputtering on cotton (1 min)	-	120	*E. coli*	3.8 × 10^6^	Vis	255	100	[88]
N-TiO_2_	F	Anodic oxidation	-	240	*E. coli*	2 × 10^6^	UV	-	33	[89]
N-TiO_2_	P	Sol–gel	0.1	360	*E. coli*	10^5^	Vis	90	-	[90]
Cr-TiO_2_			0.1						70	
Cr/N-TiO_2_			0.2						-	
N-TiO_2_	P	Hydrolisis calcination	1%	7800	*Aspergillus niger*	10^5^	Vis	-	100	[91]
N-T-TiO_2_				7200						
C-TiO_2_				7200						
Pd-CTiO_2_				5760						
V_2_O_5_/TiO_2_	P	Wet impregnation method	0.5	30	*E. coli*	10^8^	UV-C Vis	8	100	[92]
TiO_2_/Cu	F	Sputtering on polyester	-	10	*E. coli*	10^6^	Simulated sunlight	87.5	100	[93]
TiO_2_/CdS	P	Hydrothermal ultrasonication Hot injection	0.1	10	*E. coli*	10^8^	Vis	-	99	[94]
TNTZ-Cu	F	Sputtering on glass	-	75	*E. coli*	3 × 10^6^	Vis	18	100	[95]
Ti- BiOI	P	Solvothermal method	0.06	24	*E. coli*	3 × 10^7^	Vis	300	100	[96]
				45	*S. aureus*	3 × 10^6^				
CuOx-TiO_2_-PET	F	Sputtering on PET	-	20	*E. coli*	4 × 10^6^	Actinic light	-	100	[97]
TiN/TiN-Ag	F	Sputtering on polyester	-	15	*E. coli*	10^8^	Actinic light	112	100	[98]
F-ZnO	P	Sol–gel	-	360	*S. aureus*	-	Vis	150	99.99	[99]
					*E. coli*				99.87	
Fe-TiO_2_	P	Dip coating	Fixed bed	120	*E. coli*	10^6^	Solar	-	>99	[100]
Ce-ZnO	P	Precipitation	-	120	*E. coli*	1 × 10^5^	UVA	18	99.99	[101]
					*P. aeruginosa*					
PECuOx	F	Sputtering on polyester	-	15	*E. coli*	>10^6^	Sunlight	60	100	[102]
TiO_2_/Cu-PES	F	Sputtering on polyester	-	30	*E. coli*	>10^6^	Actinic light	-	100	[103]
Ce-ZnO	P	Precipitation	0.1	120	*E. coli*	10^6^	UVA	125	100	[104]
Cu-ZnO	NP	Precipitation	0.5	240	*E. coli*	10^6.5^	Simulated sunlight	300	100	[105]
ZnO/TiO_2_	NP	Sol–gel	1	20	*E. coli*	10^5^	UV	8	100	[106]
ZnCl_2_/TiO_2_, Zn(Ac)_2_/TiO_2_, Zn(NO_3_)_2_/TiO_2_ ZnSO_4_/TiO_2_	NP	Sol–gel calcination	4	120	*Candida albicans*	10^5^–10^6^	Vis	270	>95	[107]
									>87.5	
									>87.5	
									100	
ZnCl_2_/TiO_2_, Zn(Ac)_2_/TiO_2_, Zn(NO_3_)_2_/TiO_2_ ZnSO_4_/TiO_2_	NP	Sol–gel calcination	4	120	*E. coli*	10^5^–10^6^	Vis	270	>92.5	[108]
									>80	
									>90	
									100	
ZnCl_2_/TiO_2_, Zn(Ac)_2_/TiO_2_, Zn(NO_3_)_2_/TiO_2_ ZnSO_4_/TiO_2_	NP	Sol–gel calcination	4	120	*S. aureus*	10^5^–10^6^	Vis	270	>90	[109]
									>80	
									>95	
									100	

P: particle, F: film, NF: nanofiber, NT: nanotube, NTAs: nanotube arrays, S: sheets, MS: microsphere, NS: nanosheet.

**Table 3:** Polymer based photocatalysts nanocomposite for microorganism inactivation.

Photocatalyst	Form	Type	Preparation Method	Dose (g/L)	Contact Time (min)	Target	UFC/mL	Light Source	Power of Light Source (W)	Results (%)	References
PMMA/TiO_2_	F	PC	Sonication method	-	60	*E. coli*	10^5^	UV-A	-	70	[16]
PMMA/TiO_2_/SWCNTs								UV-A	-	-	[16]
PMMA/TiO_2_-TCPP								Vis	-	40	[16]
Chitosan-TiO_2_:Cu (CS-CT)	P	PFNC	Sol–gel and ultra-sonication	0.2	120	*E. coli*	3 × 10^4^	Vis	8	100	[108]
					150	*S. aureus*					
Ag-NPs@CTA	PF	PFNC	Active imprinting	0.3	120	*E. coli*	10^8^	Vis	40	99	[109]
						*S. aureus*					
						*C. albicans*					

F: film, PF: polymer-functionalized, PC: polymeric composite; PFNC: polymer-functionalized nanocomposite.

**Table 4:** Types of photocatalysts, targets, doses, endpoints, and effects.

Photocatalyst	Target	Dose (g/L)	Endpoint	Effects	Reference
Ag_3_PO_4_	*Chlorella vulgaris*	0.04	Growth inhibition	Beneficial effects	[130]
ZnO@ZnS	*Spirulina platensis*	0.025–0.4	Viability, biomass, and photosynthetic pigments	Weak effect	[131]
N- TiO_2_	*Vibrio fischeri*, *Raphidocelis subcapitata*, *Daphnia magna*	0.002 and 0.005	Growth inhibition and mortality	Weak effect	[132]
Thermally (RGOTi) and hydrogen (H_2_RGOTi)-reduced graphene oxide/TiO_2_	Zebrafish embryos	0.1, 0.2, 0.3, 0.4, 0.6, 0.8, and 1	Acutoxicity, cardiotoxicity, neurobehavioral toxicity, hematopoietic toxicity, and hatching rate	LC_50_ = 1 g/L and 0.7 g/L for H_2_RGOTi and RGOTi, respectively Decrease in body size from H_2_RGOTi Increase in eye, yolk, and pericardial size from RGOTi	[133]
Facet-dependent monoclinic scheelite BiVO_4_	Zebrafish embryos	0.02	Mortality	Weak effect	[134]
Biochar functionalized with titanium dioxide (TiO_2_)	*Mytilus galoprovincialis*	0.1	Survival, neurotoxicity, and energy metabolism	n.e.	[135]
Alumina/ZnO	Mouse	2	Gut histopathology	n.e.	[2]
Fe_2_O_3_	Wistar rats	0.02	Hearth histopathology	Cardiovascular damage	[136]
CeO/S	Laboratory rats	0.05	Biochemical effects and blood sampling	Increase in ALT and AST activity Decrease in blood cells and hemoglobin level	[137]
Zn_2_TiO_4_	Hep-2 cell line	0.3	Cytotoxicity	n.e.	[138]
Ti-nAg	Human gingival fibroblast cells	n.a.	Cytotoxicity	n.e.	[139]
Ag @chitosan–TiO_2_	Mammal cells	15.2	Cytotoxicity	Weak effect	[109]
TiO_2_:Cu	Mouse embryo fibroblast cells	2	Cytotoxicity	Beneficial effects	[108]
Multicomponent TiO_2_-based	Mouse embryo fibroblast cells Human lung cell line Human liver cell line	2.56	Cytotoxicity	EC_50_ = 0.1 g/L for mouse cell EC_50_ = 0.08 g/L for human liver cell EC_50_ => 0.3 g/L for human lung cell	[140]
O_2_-g-C_3_N_4_	Human lung cell line	0.15	Cytotoxicity	n.e.	[80]
ZnO(H)	Human lung cell line	0.08	Cytotoxicity	n.e.	[141]
CeO_2_-Fe/Cr	Aneuploid immortal keratinocyte cell line	0.025–0.1	Cytotoxicity	Cells’ viability decreased	[142]
Fe-TiO_2_	Human endothelial cells (HECV)	0.01	Cytotoxicity	Cells’ viability decreased	[143]
Fe- TiO_2_	Human endothelial cells (HECVs) Mouse macrophages (RAW 247) Hemocytes of *Mytilus galloprovincialis*	0.0001–0.001–0.01	Cytotoxicity	Cells’ viability decreased in HECVs n.e. in RAW 247 and in hemocytes of *Mytilus galloprovincialis*	[144]
Fe-TiO_2_	Human red blood cell	0.0001–0.1	Cytotoxicity	n.e.	[145]
Cd-Bi	Human colon colorectal tumor cell line	0.25–5	Cytotoxicity	Strong effect	[146]

n.a. = not available; n.e. = no effect.

## Data Availability

Not applicable.
